# Packaging of viral RNAs in virions of adenoviruses

**DOI:** 10.1186/1743-422X-6-16

**Published:** 2009-02-05

**Authors:** Li Xing, Suresh K Tikoo

**Affiliations:** 1 Vectored Vaccine Program, Vaccine & Infectious Disease Organization, University of Saskatchewan, Saskatoon, Saskatchewan, S7N 5E3, Canada; 2 Vaccinology & Immunotherapeutics Program, School of Public Health, University of Saskatchewan, Saskatoon, Saskatchewan, S7N 5E3, Canada; 3 Lady Davis Institute for Medical Research, Jewish General Hospital, Montreal, Quebec H3T 1E2, Canada

## Abstract

Earlier, we detected viral RNAs packaged in the porcine adenovirus (PAdV) -3 virions. Using Southern blot analysis, we further demonstrated that the viral RNAs were predominantly packaged in CsCl purified mature capsids (containing viral genome) than empty/intermediate capsids. Some of the packaged viral RNAs appear to be polyadenylated. Real-time reverse transcription (RT)-PCR analysis indicated that the copy number of the tested viral mRNAs encoding E1B_small _and fiber proteins was less than one per full capsid. Moreover, detection of viral RNA packaged in CsCl purified human adenovirus (HAdV) -5 virions indicates that the viral RNA packaging might be a common phenomenon in members of *Adenoviridae *family. Further quantitative analysis of viral protein, DNA, and RNA in CsCl purified mature and empty/intermediate capsids of recombinant HAdV-5 expressing enhanced green fluorescent protein indicated that the traceable viral RNA detected in empty/intermediate capsids seems associated with the presence of traceable viral genomic DNA. Taken together, our data suggest that the viral RNAs may be passively packaged in adenovirus virion during encapsidation of viral genomic DNA in cell nuclei. Thus, viral RNA packaging may be a characteristic feature of adenoviral genomic DNA encapsidation.

## Introduction

The phenomenon of encapsidation of viral RNAs was initially uncovered in members of *Herpesviridae *family [1-4]. These RNAs could be translated into proteins that would function prior to *de novo *transcription from the viral genome [1,2,5]. Alternatively, these viral RNAs might facilitate organizing the structure of the tegument domain through RNA-protein interactions during virion assembly [6] as found in retroviruses [7,8]. Moreover, the studies with human cytomegalovirus (HCMV) suggested that both the viral and the cellular RNAs were nonspecifically incorporated into the virions through interactions with some virion proteins during budding [6]. However, studies with herpes simplex virus (HSV) -1 and Kaposi's sarcoma-associated herpesvirus (KSHV) suggested that some virion RNAs were specifically incorporated into the virions [1,4].

Adenoviruses are another family of DNA viruses that infect a wide variety of mammals and birds [9]. Adenovirus is non-enveloped containing a single, linear double-stranded DNA of approximately 26–43 kb within an icosahedral capsid of 70–100 nanometer in diameter [10]. The assembly of mature adenovirus virion leads to the formation of intermediate capsids, some of which may contain little viral or cellular DNA [11-13]. Earlier, we demonstrated that viral RNAs were packaged in porcine adenovirus (PAdV) -3 virions, a non-enveloped DNA virus [14]. Another report suggested that a non-viral RNA (LacZ mRNA) transcribed from a recombinant human adenovirus (HAdV) -5 is packaged into the HAdV-5 virions [15]. In this report, we examined the incorporation of viral RNAs in mature and empty/intermediate capsids of PAdV-3 and HAdV-5. Moreover, the characteristics of packaged viral RNAs were further examined by Southern blot hybridization and real-time RT-PCR analysis.

## Methods

### Cells and Viruses

VIDO R1 cells [16] were grown in Eagle's minimum essential medium supplemented with 10% heat inactivated fetal bovine serum (FBS). The wild-type (wt) PAdV-3 (6618 strain) [17] and mutant Pav3-PL1 [18] were propagated and titrated in VIDO R1 cells. 293 cells [19] were grown in Dulbecco's modified Eagle's medium supplemented with 5% FBS. The wild-type (wt) HAdV-5 and recombinant HAV5.EGFP containing enhanced green fluorescent protein (EGFP) gene inserted in E1 region of HAdV-5 were propagated and titrated in 293 cells.

### Isolation of viral capsids

In order to obtain empty/intermediate and mature capsids, VIDO R1 cells were infected with wt PAdV-3 or mutant Pav3-PL1 at a multiplicity of infection (MOI) of 10 plaque forming units (PFUs). At 48 h post infection, the infected cells were collected and freezed-thawed three times. The cell lysates were subjected to a step gradient with 1.2 and 1.4 g of CsCl/ml and ultracentrifuged at 35,000 rpm for 2 h. Two major bands were harvested and loaded on a continuous CsCl gradient at 1.32 g/ml and centrifuged at 35,000 rpm overnight. The bands from this gradient were further purified on a third gradient and then dialyzed into phosphate-buffered saline (PBS). Similarly, HAdV-5 capsids were purified from 293 cells infected with wt HAdV-5 or recombinant HAV5.EGFP. Total RNA was isolated from purified capsids, or virus infected cells as described earlier [14].

### Electron microscopy

CsCl-purified virions were adsorbed to nickel grids. After adsorption, the grids were stained with 1% solution of phosphotungstic acid for 60 s and examined by using transmission electron microscope (Philips EM410). Photographs were taken from representative areas from each sample.

### Southern blot

The wt PAdV-3 genomic DNA was isolated from purified viral capsids as described earlier [14,18]. Viral genomic DNA or pFHAV5 plasmid DNA containing E1A-deleted HAdV-5 genome was digested with *Hind*III and loaded into 1% agarose gel. Fractionated DNAs were transferred into Gene Screen Plus hybridization transfer membranes (PerkinElmer) and probed with [^32^P]-labeled cDNA probes prepared by reverse transcribing 2 μg of RNAs isolated from purified capsids or virus infected cells in the presence of [^32^P]-labeled dCTP as described earlier [14].

### Real-time PCR

Real-time PCR was performed with Platinum Quantitative PCR SuperMix-UDG kit (Invitrogen) as instructed by manufacturer. The cDNAs were prepared by reverse transcription as described previously [14]. The synthesized cDNAs or viral genomic DNAs were used as templates in real-time PCR, using iCycler real-time PCR system (Bio-Rad). TaqMan probes for E1B_small _(5'-TTTATC AAGGTAGTAGCAG AGGCCA-3') and fiber (5'-TCCCTGGGTCCCGGTCT TTCTA ACT-3') were labeled with FAM at 5'-end and TAMRA at 3'-end, and purchased from Qiagen. The primers RTE1B1 (5'-AG TACA GGGGT CTCAGAACT-3') and RTE1B2 (5'-CTCCACAA A A ATCTCAATCA-3') are specific for PAdV-3 E1B_small_, TRFIB1 (5'-GATGGCAAGCTGG TTCTC A A-3') and TRFIB2 (5'-GGAGCTGTGACTTGCAGA CT-3') are specific for PAdV-3 fiber [20]. After incubation at 50°C for 2 min and 95°C for 2 min, the reaction was run for 45 cycles with denaturation at 95°C for 15 s, annealing and extension at 60°C for 30 s. As a negative control, RNase-treated RNA samples were also reversely transcribed and simultaneously subjected to PCR reaction. Serial 10-fold dilutions of known amount of plasmid pFPAV3 DNA (known as pPAV200)[21] containing E1B_small _and fiber genes were used as positive control to generate the standard curve. The copy numbers were calculated by converting the weight unit of double-stranded pFPAV3 DNA into the numbers of single-stranded DNA molecules. The averaged cycle threshold (Ct) values of five replicates were used to determine the relative RNA copy number.

## Results and discussion

### Preparation and characterization of PAdV-3 capsids

**Figure 1:**
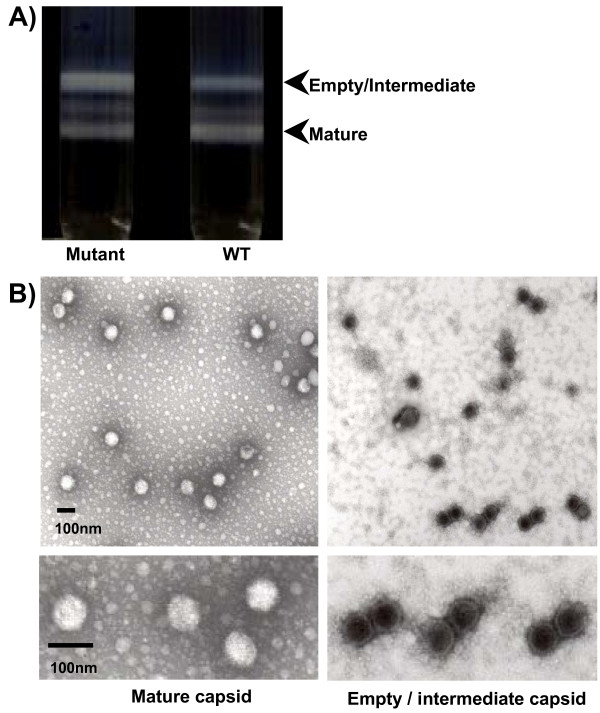
**Production of the virus capsids**. (A) Crude lysates prepared from VIDO R1 cells infected with Pav3-PL1 or wt PAdV-3 were separated by centrifugation through CsCl gradients. The position of empty/intermediate and mature capsids is indicated. (B) Electron microscopic (EM) images of CsCl-purified viral capsids.

At late times during adenovirus infection, two kinds of particles are abundant, which can be separated by CsCl gradient centrifugation [22]. The heavy particles exhibiting a density of approximately 1.32 g/ml are the mature capsids that are composed of the capsid proteins, the core proteins and the viral DNA. The lighter particles exhibiting a density of approximately 1.29 g/ml are the empty/intermediate capsids that are composed of the capsid proteins, but are usually devoid of the genomic DNA and the core proteins. In order to obtain different capsids, we chose to purify mutant Pav3-PL1, (reduced DNA packaging efficiency) [18] and wt PAdV-3. As seen in Fig. 1A, two major bands and a minor band were observed in wt PAdV-3 or in mutant Pav-3-PL1 infected cell lysate after CsCl gradient centrifugation. As expected, the upper band was heavier than the lower band in Pav-3-PL1 infected cells. The yields of both empty/intermediate and mature capsids were quantified by measuring protein concentration with Bradford protein assay (Bio-Rad).

To further characterize the integrity of empty/intermediate and mature capsids, DNA was isolated from both bands. Ethidium bromide-stained agarose gel showed that the viral genomic DNA is present predominantly in the lower bands (data not shown), confirming that the viral particles with the density of 1.32 g/ml are the mature capsids. To determine the integrity of the capsids, transmission electron microscopy was performed as described [23]. Analysis of the preparations by the electron microscope (EM) suggested that the mature and the empty/intermediate capsids of both wt PAdV-3 and mutant Pav3-PL1 are predominantly intact (Fig 1B, wild-type PAdV-3).

### Viral RNA was mainly detected in mature capsids and appeared polyadenylated

**Figure 2:**
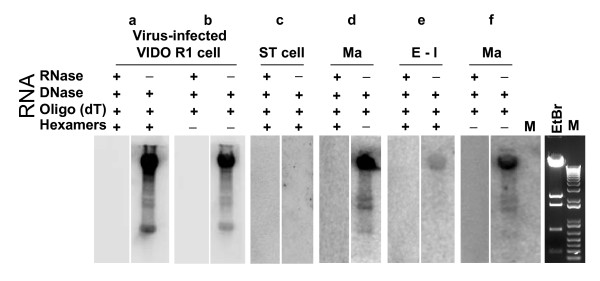
**Southern hybridization with PAdV-3 DNA**. Fractionated *Hind*III digested wt PAdV-3 genomic DNA was probed with [^32^P]-labled cDNAs generated by RT of the RNAs isolated from purified wt PAdV-3 mature capsids, empty/intermediate capsids, wt PAdV-3-infected VIDO R1 cells or uninfected ST cells as described in the text. Blots in c, d, e, and f were exposed for the same time with PhosphorImager screen (Bio-Rad). Presence (+). Absence (-). EtBr, Ethidium-bromide (EtBr) staining of *Hind*III digested PAdV-3 genomic fragments.1 Kb DNA ladder (M). Mature(Ma);Empty/Intermediate(E-I).

Earlier studies demonstrated that most of the viral RNAs were nonspecifically packaged in HCMV and KSHV particles [1,3,6]. We reasoned that if the viral RNAs were nonspecifically incorporated into PAdV-3 particles as well, the empty/intermediate capsids would be expected to contain more viral RNAs than the full capsids, since the absence of viral genome in the empty/intermediate capsids would leave extra room for RNAs. To test this hypothesis, equal amounts (based on protein concentrations) of CsCl purified empty/intermediate and mature capsids of wt PAdV-3 were treated with RNase to remove RNAs contaminated outside of viral particles before processing for the isolation of virion RNAs as previously described [14]. The isolated virion RNAs were treated with RNase-free DNase (Ambion) to eliminate the contaminated viral genomic DNA, followed by addition of 0.1 volume DNase inactivation reagent (DNA-free kit, Ambion). The RNA was also isolated from wt PAdV-3 infected cells as described previously [14]. Two micrograms of virion RNAs from each preparation were converted into [^32^P]-labeled cDNAs by reverse transcriptase II (Invitrogen) with oligo-dT and hexamers in the presence of [^32^P]-dCTP as described previously [14]. As a control, two microgram of virion RNAs from each preparation were treated with RNase before RT reaction. The resultant cDNAs were subsequently hybridized to the membrane blots containing *Hind*III-digested wt PAdV-3 genomic DNA. Positive signals were detected with radioactive probes made from the RNAs isolated from wt PAdV-3 infected cells (Fig. 2, panel a). Similarly, positive signals were also detected with radioactive probes made from the virion RNA isolated from the mature capsids (Fig. 2, panel d). No signal could be detected with radioactive probes made from the RNase treated RNAs isolated from PAdV-3 infected cells (Fig. 2, panel a) or purified mature capsids (Fig 2, panel d) indicating that the positive signals were detected due to labeled probes generated from the viral RNAs. These results confirmed our earlier observation that the viral RNAs are packaged in the mature capsid [14]. Compared with mature capsids (Fig. 2, panel d), a weak signal was detected with radioactive probes made from the RNAs isolated from the empty/intermediate capsids (Fig. 2, panel e), suggesting that empty/intermediate capsids might contain only trace amounts of viral RNAs. The trace virion RNAs detected in the empty/intermediate capsids might be the RNAs packaged nonspecifically in empty/intermediate capsids. Taken together, the results indicated that the viral RNAs were mainly packaged in the mature capsids containing viral genomic DNAs.

To further characterize the viral RNAs packaged in PAdV-3 virions, reverse transcription (RT) of the RNAs from the mature capsids was primed with only oligo-dT of 12–16 nucleotides (nt), which is specific for mRNAs containing polyA tail. As a positive control, 2 μg of total RNA isolated from wt PAdV-3-infected VIDO R1 cells at late times post infection was reversely transcribed in the presence of [^32^P]-dCTP as described above. As a negative control, 2 μg of total RNA from swine testis (ST) cells was reversely transcribed in the presence of [^32^P]-dCTP. As expected, radiolabeled probes made from the RNAs isolated from uninfected ST cells did not produce any positive signals in Southern blot (Fig. 2, panel c). In contrast, radiolabeled probes made from RNAs isolated from wt PAdV-3-infected VIDO R1 cells resulted in strong signals, indicating that PAdV-3 specific RNAs were present in cellular RNA preparations (Fig. 2, panel b). No signal could be detected with radiolabeled probes made from the RNase treated RNA isolated from wt PAdV-3-infected VIDO R1 cells (Fig. 2, panel b). Compared with the oligo-dT and hexamer-doubly primed RT reaction (Fig. 2, panel a), the oligo-dT primed RT reaction displayed weaker positive signals (Fig. 2, panel b). Compared to the results obtained with total RNAs from the virus infected VIDO R1 cells, the oligo-dT primed RT of virion RNAs from purified mature capsids also produced weaker positive signals (Fig. 1B. panel f) than RT primed with both oligo-dT and hexamer (Fig. 2. panel d). These results suggested that some viral RNAs packaged in the virions appear to be polyadenylated.

### Viral RNA was packaged at a low copy number into PAdV-3 mature capsids

To quantify the RNAs packaged in the virions, we performed real-time RT-PCR with Platinum Quantitative PCR SuperMix-UDG kit (Invitrogen). We choose to quantitate RNAs specific for an early region gene (E1B_small_) and a late region gene (fiber) of PAdV-3. The viral genomic DNA and RNA were isolated from the same pool of purified mature capsids. Viral DNA was used as a template in real-time PCR to determine the total number of viral particles containing viral genome. The isolated RNAs with or without RNase treatment were reversely transcribed with the oligo-dT and hexamer as described above before using as templates in the real-time PCR to determine the RNA copy number based on the cycle threshold (Ct) values. Real-time PCR was performed by using Cycler real-time PCR system (Bio-Rad) as described in Methods.

**Figure 3:**
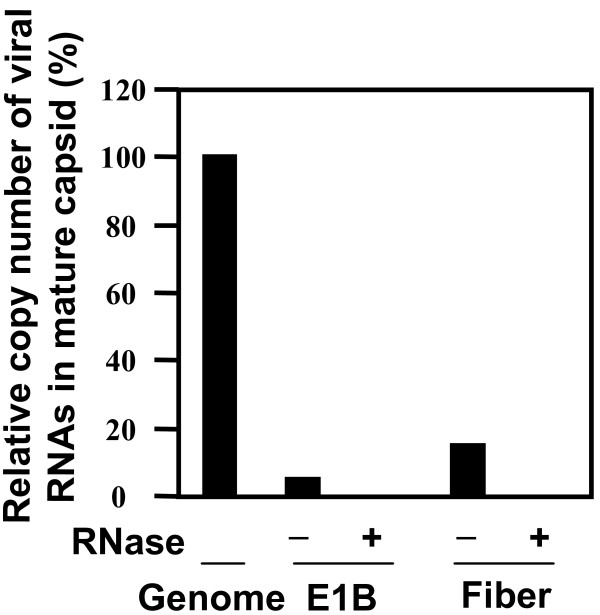
**Quantitation of viral RNAs by real-time PCR**. Viral genomic DNA and RNAs were isolated from the same pool of purified mature capsids. RNAs with (+) or without (-) RNase treatment were reversely transcribed with oligo-dT and hexamers, and then used as templates in the real-time PCR with TaqMan probes. The calculated copy number of the mRNAs of E1B_small _and fiber is shown as percentage relative to the copy number of the viral genomic DNA. The averaged cycle threshold (Ct) values of 5 replicates were used to determine the relative RNA copy number.

The RNA copy number was finally normalized with the total number of genome-containing particles designated as 100%, and shown as relative percentage. The copy number of E1B_small _and fiber RNAs in mature capsids were 5% and 15% respectively (Fig. 3) of the number of genome-containing particles, indicating that no more than one copy of E1B_small _or fiber RNA was packaged in a mature capsid. Earlier report also suggested that at the most one copy of a reporter mRNA produced from recombinant HAdV-5 was packaged in the viral particles [15].

### Human adenovirus type 5 particles also contain viral RNAs

**Figure 4:**
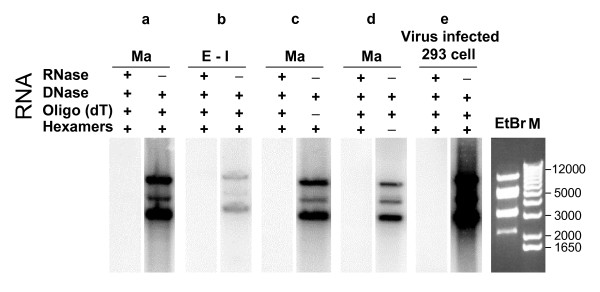
**Southern hybridization with HAdV-5 DNA**. Fractionated *Hind*III digested pFHAV5 DNA was probed with [^32^P]-labeled cDNAs generated by reverse transcription of RNAs isolated from purified wt HAdV-5 mature capsids, empty/intermediate capsids, or wt HAdV-5-infected 293 cells. Blots in a, b, c, d, and e were exposed for the same time with PhosphorImager screen (Bio-Rad). Presence (+). Absence (-). Ethidium-bromide (EtBr) staining of *Hind*III digested pFHAV5 DNA fragments. 1 Kb DNA ladder (M). DNA band sizes are indicated in bps on the right. Mature(Ma); Empty/Intermediate(E-I).

To determine if viral RNAs were packaged into HAdV-5 virions, both empty/intermediate and mature capsids were purified from wt HAdV-5 infected 293 cells [19] using the procedure described earlier [14]. Equal amounts (based on protein concentrations) of CsCl purified HAdV-5 capsids were treated with RNase and then extracted with Trizol reagent (Invitrogen) to isolate the virion RNAs. Two micrograms of virion RNAs isolated from mature or empty/intermediate capsids were reversely transcribed by reverse transcriptase II in the presence of [^32^P]-dCTP. The synthesized [^32^P]-labeled cDNAs were hybridized to *Hind*III-digested plasmid pFHAV5 that contains E1A-deleted HAdV-5 genome. Positive signals were detected with radioactive probes made from the RNAs isolated from wt HAdV-5 infected 293 cells (Fig. 4, panel e). Similarly, positive signals were also detected with radioactive probes made from the virion RNA isolated from mature capsids (Fig. 4, panels a, c, and d). No signal could be detected with radioactive probes made from the RNase treated RNAs isolated from wt HAdV-5 infected 293 cells (Fig. 4, panel e) or mature capsids (Fig. 4, panels a, c, and d), indicating that positive signals were detected due to labeled probes generated from viral RNAs. Similar to wt PAdV-3 (Fig. 2, panel b), a weak signal was detected with radioactive probes made from the RNA isolated from empty/intermediate capsids (Fig. 4, panel b). The oligo-dT/hexamer (Fig 4, panel a), hexamers alone (Fig. 4, panel c), and the oligo-dT alone (Fig. 4, panel d)-primed RT of the virion RNAs from mature capsids all resulted in positive signals in Southern blot analysis. The data indicated that wt HAdV-5 particles containing the genomic DNA also contain more viral RNA and that part of the virion RNAs are potentially polyadenylated. The phenomenon of packaging of viral RNAs in adenoviral particles is not unique to PAdV-3 that naturally infects pigs.

### Viral RNA packaging appears to be associated with adenovirus genomic DNA encapsidation

**Figure 5:**
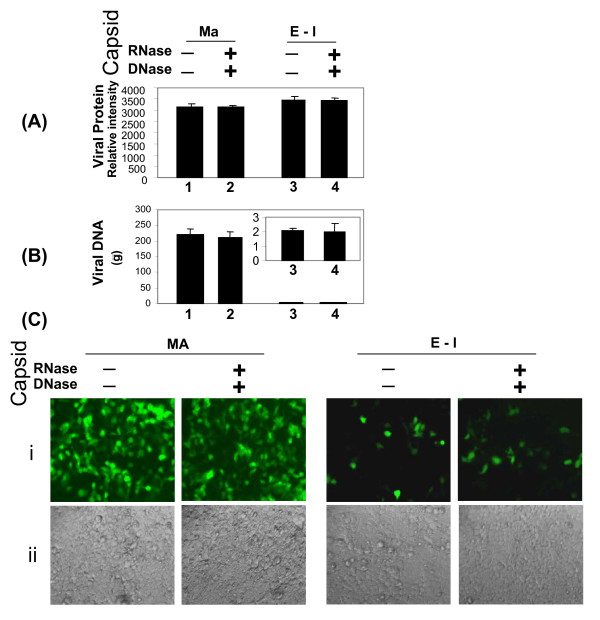
**Analysis of viral protein and DNA in HAV5.EGFP capsids**. **(A) **Proteins from lysates of RNase/DNase treated or untreated mature and empty/intermediate capsids were separated by 10% SDS-PAGE, and stained by SYPRO Ruby protein stains. A 100 kDa hexon protein band was used for determining the capsid protein concentration using Kodak IM Network software. **(B) **Total yields of DNAs isolated from mature and empty/intermediate capsids with or without RNase/DNase treatment. (**C**) EGFP expression. 293 cells were transduced by 10 fold serially diluted mature and empty/intermediate capsids with or without RNase/DNase treatment. At 48 h post transduction, cells were analysed for EGFP expression by a fluorescent microscope (i) EGFP, (ii) Phase contrast. Presence (+). Absence (-). Mature (Ma); Empty/Intermediate (E-I).

To determine why the viral RNAs are predominantly detected in adenoviral mature capsids, the RNA and DNA were quantitatively analyzed in CsCl purified mature and empty/intermediate capsids of HAV5.EGFP. CsCl purified mature and empty/intermediate HAV5.EGFP capsids were mock treated or treated with RNase A (Ambion) and DNase I (Invitrogen) at room temperature. After 4 h incubation at room temperature, 10 μl of each sample was dissolved in RIPA buffer [16,21]. Proteins from capsid lysates were separated by sodium dodecyl sulphate – polyacrylamide gel electrophoresis (SDS-PAGE) and stained with SYPRO Ruby protein stains (Bio-Rad) overnight. Quantitative analysis of adenoviral hexon protein (Fig. 5A) suggested that the RNase/DNase treatment did not affect the virion protein concentration.

Since both mature and empty/intermediate capsids may contain viral/cellular DNA [11-13], initially we quantitated the viral DNA in these capsids. Equal amounts (based on protein concentrations) of CsCl purified capsids were treated with RNase A and DNaseI as described above. The samples were further treated with 100 μg/ml proteinase K (Invitrogen) at 50°C for 2 h, before extracting sequentially with phenol and chloroform. The DNA was precipitated by isopropanol and dissolved in RNase A containing TE buffer (10 mM Tris-Cl, 1 mM EDTA, pH8.0). Quantitative analysis suggested that mature capsids yielded 100 times more DNA than empty/intermediate capsids irrespective of the RNase/DNase treatment (Fig. 5B). The significant difference in the amount of DNA obtained from mature and empty/intermediate capsids is consistent with the general suggestion that mature capsid contains mature viral genomic DNA and empty/intermediate capsid may contain little premature viral DNA [11-13]. Moreover, DNA in mature and empty/intermediate capsids is not accessible to DNase/RNase treatment of the intact capsids. To confirm this further, we examined the expression of EGFP in 293 cells infected with equal amounts of mature or empty/intermediate capsid samples with or without RNase/DNase treatment. As seen in Fig. 5C, EGFP expression was detected in cells infected with mature or empty/intermediate capsids. However, empty/intermediate capsids transduced 10^4 ^fold less cells than the mature capsids. These results suggested that empty/intermediate capsids may contain premature viral and/or cellular DNA. Earlier reports have suggested that some of the intermediate capsids may contain left end of the adenovirus genome [11,12].

**Figure 6:**
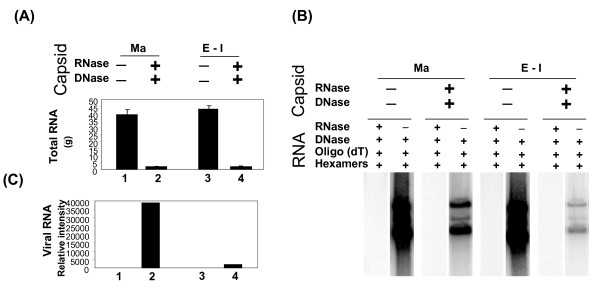
**Analysis of RNA in HAV5.EGFP capsids with or without RNase/DNase treatment**. **(A) **Total yields of RNAs isolated from mature and empty/intermediate capsids with or without RNase/DNase treatment. **(B) **The [^32^P]-labeled cDNAs were made by reverse transcription of 2 μg, RNase-free DNase treated RNAs from mature and empty/intermediate capsids with or without RNase/DNase treatment and hybridized to *Hind*III-digested pFHAV5, which contains E1A-deleted HAdV-5 genome. RT was primed by oligo-dT/hexamers.**(C) **Viral RNAs detected in RNAs from mature (Ma) and empty/intermediate (E-I) capsids after RNase/DNase treatment in Southern hybridization in panel B were quantitated by using PhosphorImager software.

Next, we quantified the RNAs present in the capsids. RNA was extracted from equal amounts (based on protein concentrations) of CsCl purified HAV5. EGFP mature and empty/intermediate capsids as previously described [14]. Equal amount (42 μg) of total RNAs were isolated from both mature and empty/intermediate capsids without RNase/DNase treatment (Fig. 6A). However, RNase/DNase treatment reduced the total RNA yields (only 2 μg) of both mature or empty/intermediate capsids by 21 fold (Fig. 6A). These results indicated that a large amount of RNAs bound to the capsid surface could not be removed by CsCl ultracentrifugation. To analyze the amount of viral RNAs present in the total RNAs isolated from mature and empty/intermediate capsids, 2 μg of RNA isolated from mature or empty/intermediate capsids without RNase/DNase treatment was reversely transcribed by reverse transcriptase II (Invitrogen) in the presence of [^32^P]-dCTP. Synthesized [^32^P]-labeled cDNAs were hybridized to *Hind*III-digested plasmid pFHAV5 containing E1A-deleted HAdV-5 genome. As expected, the cDNA probes resulted in positive signals as strong as the cDNA probes made from wt HAdV-5 infected 293 cell RNA (Fig. 6B). This is consistent with the suggestion that CsCl purification does not remove the RNAs bound to capsid surfaces.

To determine the identity of the RNAs isolated from RNase/DNase treated mature and empty/intermediate capsids, cDNA probes were synthesized using 2 μg of RNA isolated from mature or empty/intermediate capsids with RNase/DNase treatment and hybridized to *Hind*III-digested plasmid pFHAV5 containing E1A-deleted HAdV-5 genome. As seen in (Fig. 6B), these cDNA probes also resulted in positive signals, confirming that there are viral RNAs present inside the capsids. Although the total RNA yields from mature and empty/intermediate cpasids with RNase/DNase treatment appeared similar (Fig. 6A), the quantitative analysis indicated that the mature capsids contain more (30 fold) viral RNAs than empty/intermediate capsids (Fig. 6C). It is possible that the empty/intermediate capsids contain more cellular RNA. However, absence of detection of cellular mRNAs of house-keeping genes such as actin in virion RNAs [14] raises the possibility that the majority of the cellular RNAs isolated from mature and empty capsids might be rRNAs.

Although the virion RNAs were detected in the mature capsids, the low copy number of examined RNAs suggests that the viral RNAs might be packaged in a passive way by accompanying the viral genomic DNA at the stage of DNA encapsidation. So far, two models for adenovirus assembly have been proposed on the basis of pulse-chase analysis and the study of mutant viruses. The traditional model is that the adenovirus assembly begins with the formation of empty capsids, followed by the insertion of viral DNA into preformed empty capsids [24-26]. However, recently proposed model suggests that the capsids are assembled around the viral genomic DNA [27,28]. The insertion of viral genome into the preformed empty capsids more likely would separate the synthesized RNAs from viral genome, resulting in missing of the viral RNAs in full capsids containing the viral genome. Thus, a mechanism of adenovirus assembly where packaging of virion RNAs with genomic DNA is coordinated with capsid formation appears to be plausible.

## Competing interests

The authors declare that they have no competing interests.

## Authors' contributions

LX designed and carried out the experiments, and helped to analyze the data and draft the manuscript. SKT helped to design the study, help in interpretation of the data and edit the manuscript. Both authors read, made corrections and approved the final manuscript.
